# In-vivo data-driven parcellation of Heschl’s gyrus using structural connectivity

**DOI:** 10.1038/s41598-022-15083-z

**Published:** 2022-07-04

**Authors:** Hyebin Lee, Kyoungseob Byeon, Bo-yong Park, Sean H. Lee, Hyunjin Park

**Affiliations:** 1 Department of Electrical and Computer Engineering, Sungkyunkwan University, Suwon, South Korea; 2 Center for Neuroscience Imaging Research, Institute for Basic Science, Suwon, South Korea; 3 Department of Data Science, Inha University, Incheon, South Korea; 4 Department of Neuroscience, Max Planck Institute for Empirical Aesthetics, 600322 Frankfurt, Germany; 5 Brain Imaging Center, Goethe University, Frankfurt, Germany; 6 School of Electronic and Electrical Engineering, Sungkyunkwan University, Suwon, South Korea

**Keywords:** Computational biology and bioinformatics, Neuroscience

## Abstract

The human auditory cortex around Heschl’s gyrus (HG) exhibits diverging patterns across individuals owing to the heterogeneity of its substructures. In this study, we investigated the subregions of the human auditory cortex using data-driven machine-learning techniques at the individual level and assessed their structural and functional profiles. We studied an openly accessible large dataset of the Human Connectome Project and identified the subregions of the HG in humans using data-driven clustering techniques with individually calculated imaging features of cortical folding and structural connectivity information obtained via diffusion magnetic resonance imaging tractography. We characterized the structural and functional profiles of each HG subregion according to the cortical morphology, microstructure, and functional connectivity at rest. We found three subregions. The first subregion (HG_1_) occupied the central portion of HG, the second subregion (HG_2_) occupied the medial-posterior-superior part of HG, and the third subregion (HG_3_) occupied the lateral-anterior-inferior part of HG. The HG_3_ exhibited strong structural and functional connectivity to the association and paralimbic areas, and the HG_1_ exhibited a higher myelin density and larger cortical thickness than other subregions. A functional gradient analysis revealed a gradual axis expanding from the HG_2_ to the HG_3_. Our findings clarify the individually varying structural and functional organization of human HG subregions and provide insights into the substructures of the human auditory cortex.

## Introduction

The auditory cortex handles both sensory processing of hearing and language-related tasks in the auditory systems of many vertebrates^1^. The structure of the auditory cortex in nonhuman primates is well established and is divided into several substructures (i.e., core, belt, and parabelt) based on microstructural and functional information^2^. In humans, the Heschl’s gyrus (HG) is comparable and it contains the primary auditory cortex located on the inferior surface of the lateral fissure^3,4^. HG structure and underlying auditory processing mechanism are more complex than those of nonhuman primates^5^. This is due to the differences in the HG structure among individuals induced by neural plasticity or innate factors^6–8^. Except for the single HG, the most common subtypes in the individual gyrification pattern of HG are related to the bifurcations such as common stem duplications (partially separated) and full posterior duplications (fully separated). These factors lead to increased inter-individual variability in anatomy and function in HG^4^. This heterogeneity makes it difficult to have a group-level parcellation in the human HG and made it hard to intuitively compare the structural and functional properties of the auditory cortex between nonhuman primates and humans. Thus, an individualized parcellation of the human auditory cortex is needed to assess its structural and functional profiles across individuals.

Neuroimaging tools particularly magnetic resonance imaging (MRI), together with machine-learning techniques, allow the subregions of the auditory cortex to be investigated in vivo including a detailed description of the medial portion of HG among others^4,9^. Especially, one study proposed an automatic method to segment the HG accounting for the ambiguity of the HG using structural MRI enabling accurate downstream analysis^8^. Brain parcellation is a technique that divides a given region of interest (ROI) into subregions (i.e., clusters) with similar spatial, temporal, or geometric patterns. In previous studies, the parcellation approach was applied to the human brain to identify distinct subregions of the thalamus, frontal cortex, and motor area, along with other regions^10–12^. Majority of studies subdivided human HG using its morphology, MRI informed microstructure (e.g., myelin density, cortical thickness), and functional responses. Using cortical morphology based on MRI, the human auditory cortex was divided into several distinct parcels, including the planum polare, transverse temporal gyrus or HG, and planum temporale^13^. Furthermore, some studies showed evidence that the medial portion of HG includes the human primary auditory cortex, which corresponds to the core area of nonhuman primates^14,15^. However, the results from frequency response from tonotopic study indicated that there may be several subregions that cannot be divided by the cortical morphology along^3^. The studies indicated that their spatial patterns have low reproducibility owing to the heterogeneity of the brain structure and sensitivity to the frequency response across individuals from tonotopic maps^3^. Based on these points, we suggest that individualized HG parcellation may require additional information other than morphology or functional response. One study estimated intrinsic connectivity in the human HG and reported that there are several parts inside HG having distinct connection patterns to each other^16^. Further, a recent surgical study reported that HG is a hub region containing intersecting fiber tracts connected to many different adjacent regions^17^. Thus, we hypothesized that fiber information derived from diffusion-weighted MRI tractography can be used to study individual-level HG subregions and the result may contain several distinct subregions beyond the core-like area of nonhuman primates.

**Figure 1:**
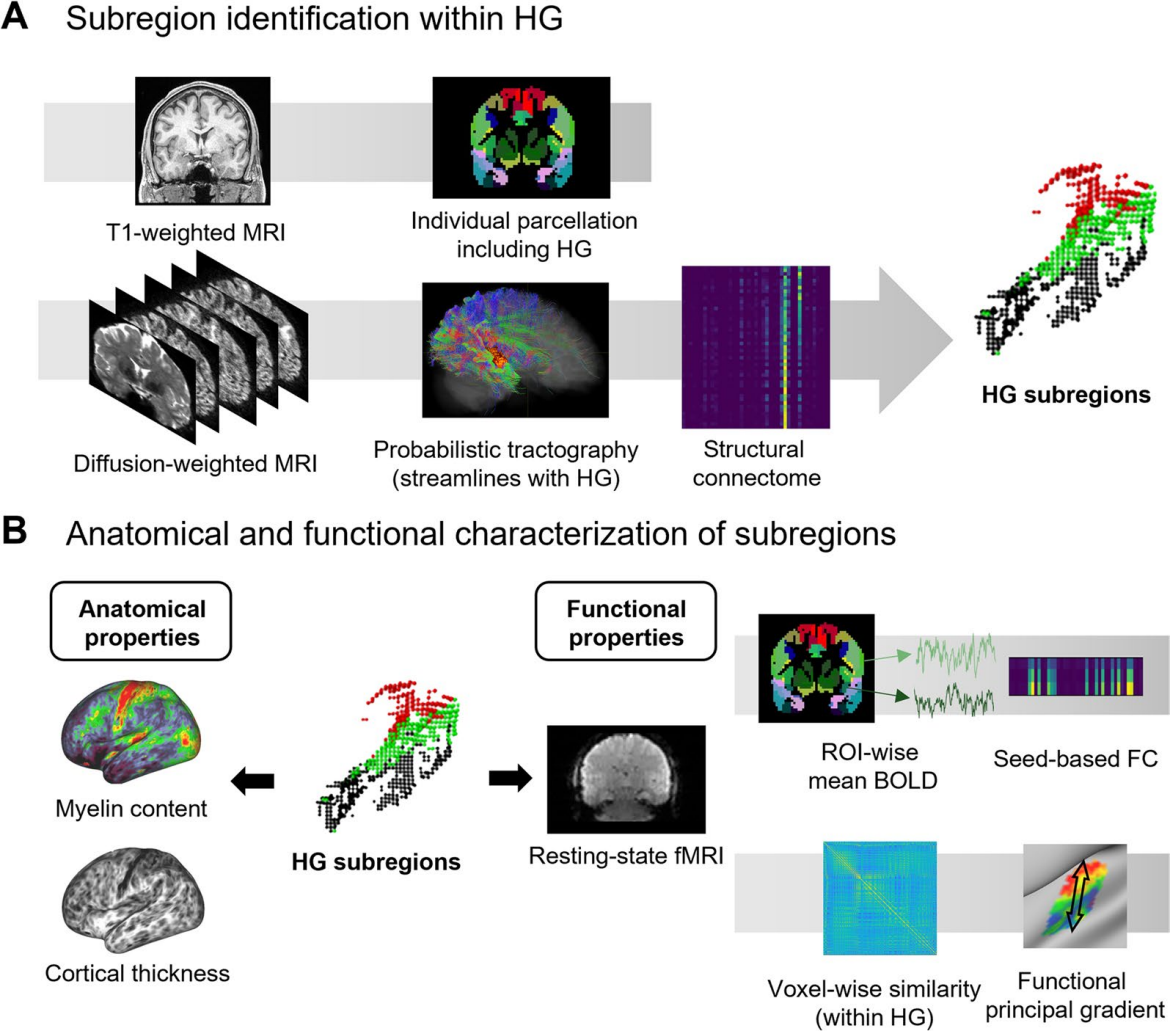
Flowchart of the study. (**A**) Individual-level parcellation was performed using T1-weighted MRI to define the HG. Subregions were identified via clustering approaches using seed-based structural connectivity information with the seed of the individual HG. (**B**) We characterized anatomical (i.e., myelin contents and cortical thickness) and functional properties (i.e., functional connectivity and gradient) of the HG subregions.

In this study, we investigated the subregions of the HG in humans via data-driven machine-learning techniques without any bias towards the number of subregions at the individual level, using imaging features of cortical folding and structural connectivity derived from diffusion MRI tractography^18^. Additionally, we characterized the structural and functional profiles of the subregions of the HG according to the cortical morphology and microstructure, as well as the functional connectivity at rest. A flowchart of the study is presented in Fig. 1.

## Methods

### Participants and imaging data

We studied 207 young healthy adults from the Human Connectome Project (HCP) database^19^, which provided full demographic information as well as multimodal imaging data of T1-weighted structural MRI, diffusion-weighted imaging (DWI), and resting-state functional MRI (rs-fMRI) with sufficient data quality. We visually inspected the data quality, with a focus on whether the data showed a clear boundary of the cortical surface in the temporal lobe in all the imaging modalities. This retrospective study was approved by the Institutional Review Board (IRB) of Sungkyunkwan University and performed in full accordance with the local IRB guidelines. All the participants provided informed consent.

All imaging data were obtained using a Siemens 3 T Skyra scanner. T1-weighted structural data were scanned using a magnetization-prepared rapid gradient-echo (MPRAGE) sequence (repetition time [TR] = 2400 ms, echo time [TE] = 2.14 ms, inversion time [TI] = 1000 ms, flip angle = 8°, field-of-view [FOV] = 224 × 224 mm^2^, voxel resolution = 0.7 mm^3^, 176 slices). The DWI was performed using a spin-echo echo planar imaging (EPI) sequence (TR = 5520 ms, TE = 89.5 ms, flip angle = 78°, FOV = 210 × 180 mm^2^, voxel resolution = 1.25 mm^3^, 111 slices, number of diffusion directions = 270, b-values = 1000, 2000, and 3000 s/mm^2^). The rs-fMRI was performed using a gradient-echo EPI sequence (TR = 720 ms, TE = 33.1 ms, flip angle = 52°, FOV = 208 × 180 mm^2^, voxel resolution = 2 mm^3^, 72 slices, and 1400 volumes).

### Data preprocessing

The multimodal MRI data were subjected to the HCP minimal preprocessing pipelines^20–23^. In brief, the gradient nonlinearity distortion, intensity inhomogeneity, and readout distortion were corrected for the structural MRI data. T1-weighted and T2-weighted data were co-registered, and the inverse intensities from T1- and T2-weighting were used for bias field correction. The processed data were registered onto the MNI152 standard space, and white and pial surfaces were generated^24–26^. The mid-thickness surface was generated by averaging the white and pial surfaces and was used to generate a spherical surface, which was registered onto the Conte69 template. For diffusion MRI, after b0 intensity normalization, the eddy current-induced field inhomogeneities and head motion were corrected. The corrected data were resampled onto a 1.25-mm native space. The rs-fMRI data underwent gradient distortions and head motion correction, registration to T1-weighted structural data, subsequent registration onto the MNI152 standard space, magnetic field bias correction, skull removal, and intensity normalization across the 4D volumes. Nuisance components were removed using FIX^27^, and the preprocessed volume data were mapped to the standard grayordinate space using a cortical ribbon-constrained volume-to-surface mapping algorithm.

### HG subregion identification

To identify the subregions within the HG, we utilized the structural connectivity derived from diffusion MRI tractography. Specifically, we performed tractography using diffusion MRI data preprocessed via MRtrix3^28^. For anatomically constrained tractography^29^, different tissue types of the cortical and subcortical gray matter, white matter, and cerebrospinal fluid were segmented from the preprocessed T1-weighted image. The multi-shell and multi-tissue response functions were estimated^30^ and constrained spherical deconvolution was performed to estimate the fiber orientation distributions^31^. Streamline tractography was performed using the iFOD2 algorithm^32^ by randomly placing 10,000 seeds per voxel within an individually defined HG region, according to the Desikan–Killiany atlas^33^. To mitigate the ambiguity of the boundary of the HG, we dilated the initial HG region 2.1 mm in the direction of the STG and insula. We estimated the seed-to-whole brain structural connectivity by mapping the intrahemispheric streamlines between the voxels in the dilated HG region and the rest of the ROIs defined in the Desikan–Killiany atlas. To identify the subregions of the HG, we then applied K-means clustering, whereby the subregion boundaries were determined using the Euclidean distance-based spatial proximity. We determined the optimal number of clusters by a data-driven measure of inertia, i.e., the sum of squared distances of samples to their closest cluster center, varying K from 2 to 10 for all the participants. According to the Kneedle method^38^, we selected the K that exhibited the steepest inertia reduction as the optimal number of clusters. A step-by-step protocol to identify subregions is given in the supplement (Supplementary Fig. 1).

### Structural properties of HG subregions

To characterize the structure of the identified subregions within the HG, we assessed the intracortical microstructure measured by the T1-/T2-weighted imaging contrast ratio, which is a proxy for intracortical myelin^39,40^, and the cortical thickness calculated using MRI. We averaged the myelin and thickness values within each subregion and conducted paired t-tests for each pair of subregions. Multiple comparisons were corrected using the false-discovery rate (FDR < 0.05)^39^.

### Functional connectivity profiles of HG subregions

Next, we investigated whether these subregions exhibited distinct functional connectivity profiles. Using rs-fMRI data, we constructed a functional connectivity matrix by computing the Pearson’s correlation of the time series between seed voxels within each subregion and the rest of the ROIs defined in the Desikan–Killiany atlas. To obtain a scale-free topology^34–36^, we applied soft thresholding to the correlation coefficients using the following formula: ${\left(\frac{r+1}{2}\right)}^{\beta }$, where *r* is the correlation coefficient and *β* is the scale-free index, which was set as six^37,38^. For each target region, we performed paired t-tests to compare the seed-to-whole brain functional connectivity between each pair of HG subregions and corrected multiple comparisons using the FDR^39^.

### Functional gradient within HG

We investigated the gradually changing functional connectivity patterns along the cortex within the HG using manifold learning techniques to determine whether a hierarchical axis existed within the HG. Following a recently proposed method^41^, we first prepared the (i) vertex within HG-by-time and (ii) vertex outside HG-by-time matrices. We normalized the time series for each vertex and applied singular value decomposition (SVD) to the vertex outside the HG-by-time matrix to reduce the dimensionality of the spatial axis. We calculated the Pearson’s correlation between vertices within the HG-by-time matrix and SVD-transformed matrix and then computed the cosine similarity, yielding a square similarity matrix whose size was the number of vertices within the seed region (i.e., HG). We then conducted a principal component analysis to obtain low-dimensional components (hereinafter referred to as “gradients”). Using Procrustes alignment, individual gradients were aligned onto the reference gradient calculated by applying principal component analysis to the stacked gradients of all subjects^42–44^. We compared the first functional gradient that explained most of the variance in the input similarity matrix across the subregions.

## Results

### Parcellation of HG subregions

**Figure 2:**
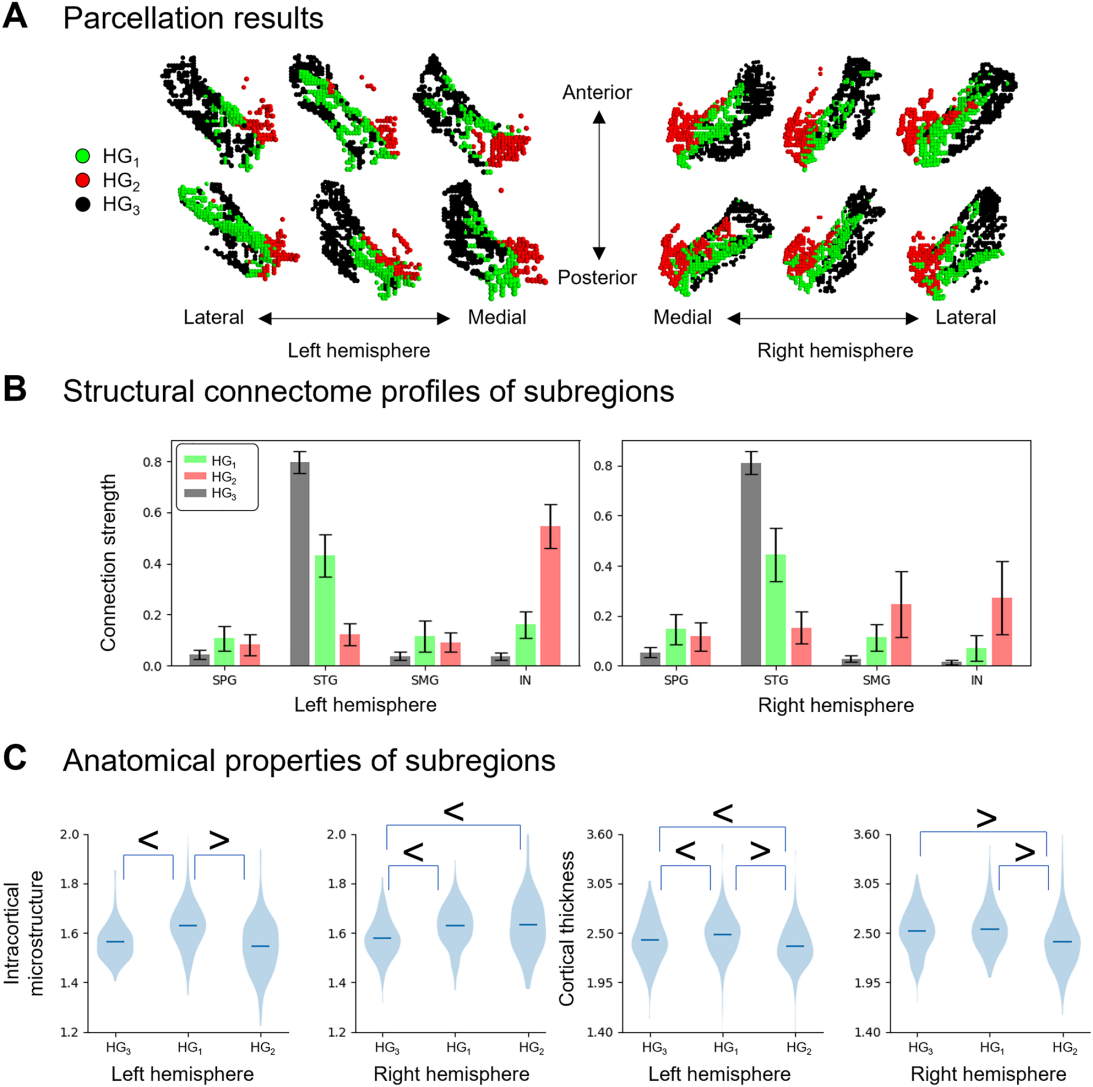
Subregions of the HG. (**A**) We identified three subregions within the HG: the HG_1_ (green), HG_2_ (red), and HG_3_ (black). The results of six randomly selected participants were visualized. (**B**) Seed-based structural connectivity profiles of each subregion to the SPG, STG, SMG, and insula (IN) are shown in box plots. The error bar indicates the standard deviation across individuals. (**C**) Intracortical microstructure and cortical thickness were plotted for each subregion, and significant differences between subregion pairs were marked with inequality signs ($${p}_{FWE}<0.05)$$. HG, Heschl’s gyrus; FWE; family-wise error.

The optimal number of clusters by computing the inertia was three from the inertia profile (Supplementary Fig. 2). By leveraging unsupervised machine learning, we identified three subregions within the HG for both hemispheres: (i) the first subregion (HG_1_) occupied the central portion of HG, (ii) the second subregion (HG_2_) occupied the medial-posterior-superior part of HG, and iii) the third subregion (HG_3_) occupied the lateral-anterior-inferior part of HG (Fig. 2A, Supplementary Fig. 3). The HG_1_ was the largest subregion of the HG (left hemisphere: 46.3% ± 10.8%; right hemisphere: 44.6% ± 10.0%), followed by the HG_3_ and then the HG_2_ (Supplementary Fig. 4). Comparing the seed-based structural connectivity strengths among the subregions revealed significant differences in the superior parietal gyrus (SPG), STG, supramarginal gyrus (SMG), and insula (IN) (p_FWE_ < 0.05; FWE, familywise error; Fig. 2B). Among the three subregions, the HG_3_ exhibited the strongest connectivity with the STG and HG_2_, which were located in the opposite direction, and had the strongest connectivity with the IN and SMG (only in the right hemisphere), and the HG_1_ exhibited moderate connection strengths with these regions. These findings suggest distinct structural connectivity profiles among the subregions, which necessitate the investigation of HG subregions.

### Microstructural and morphological profiles of subregions

To determine the macroscopic and microscopic properties of the HG subregions, we assessed the intracortical microstructure using the T1w/T2w ratio and cortical thickness of the subregions. The myelin density was higher in the HG_1_ than in the other subregions (Fig. 2C), indicating stronger laminar differentiation in the HG_1_, whereas the HG_3_ and HG_2_ exhibited relatively unclear lamination and agranular cortical profiles. The cortical thickness exhibited consistent patterns, supporting our finding that the HG_1_ may have higher cell densities.

### Seed-based functional connectivity of subregions

**Figure 3:**
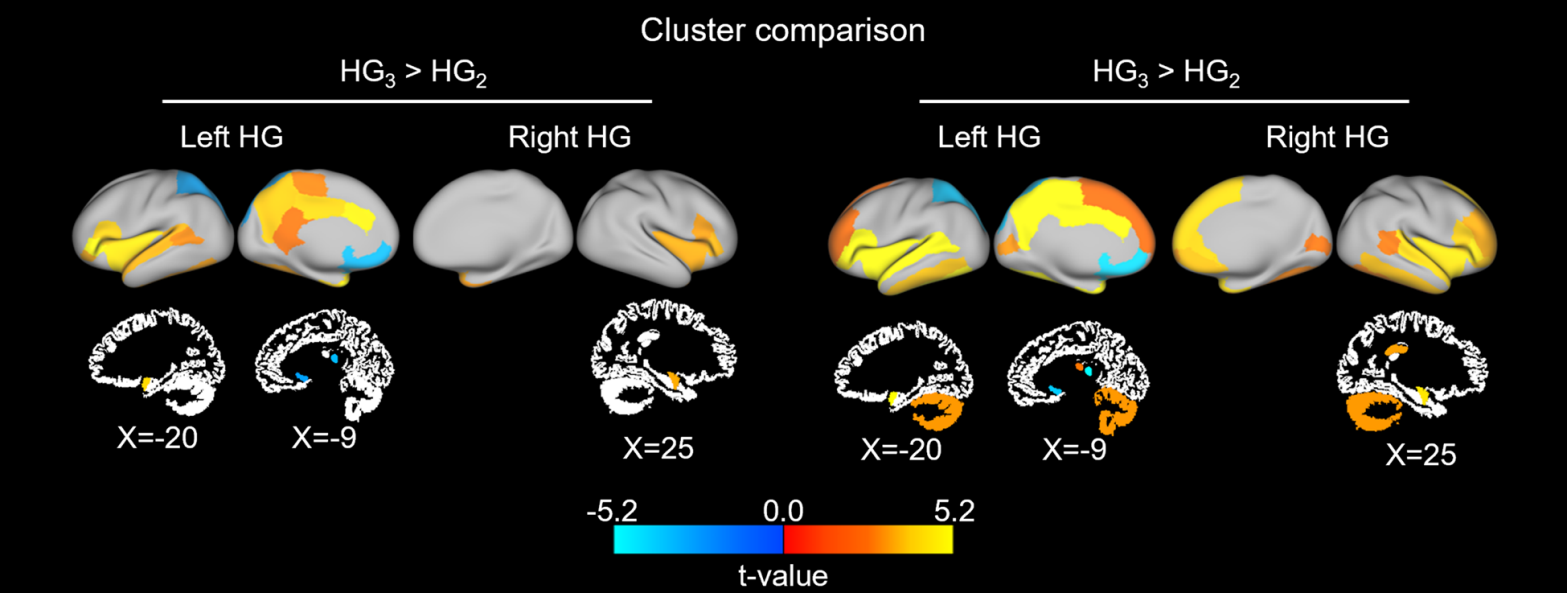
Differences in seed-based functional connectivity between subregion pairs. We visualized t-values that indicated significant differences in functional connectivity between the HG_3_ and the HG_1_/HG_2_ in the intra-hemispheric cortical and subcortical regions. We did not report the differences between the HG_1_ and HG_2_, as there were no significant differences in functional connectivity. ROI, region-of-interest.

Furthermore, we assessed the seed-based functional connectivity of each subregion to the whole brain to investigate the functional profiles of the HG subregions. The whole-brain seed-based functional connectivity for the three subregions was reported in the supplement (Supplement Fig. 5). Compared with the HG_1_ and HG_2_, we found stronger connections between the HG_3_ and the insular cortex, precuneus, and cingulate regions, as well as the amygdala and cerebellum, whereas weaker connections were observed with the superior parietal and orbitofrontal cortices (Fig. 3). The HG_1_ and HG_2_ groups did not exhibit any statistically significant differences in connectivity. These results indicate that the HG_3_ is more strongly connected to higher-order heteromodal association and paralimbic areas.

### Functional gradients within HG

**Figure 4:**
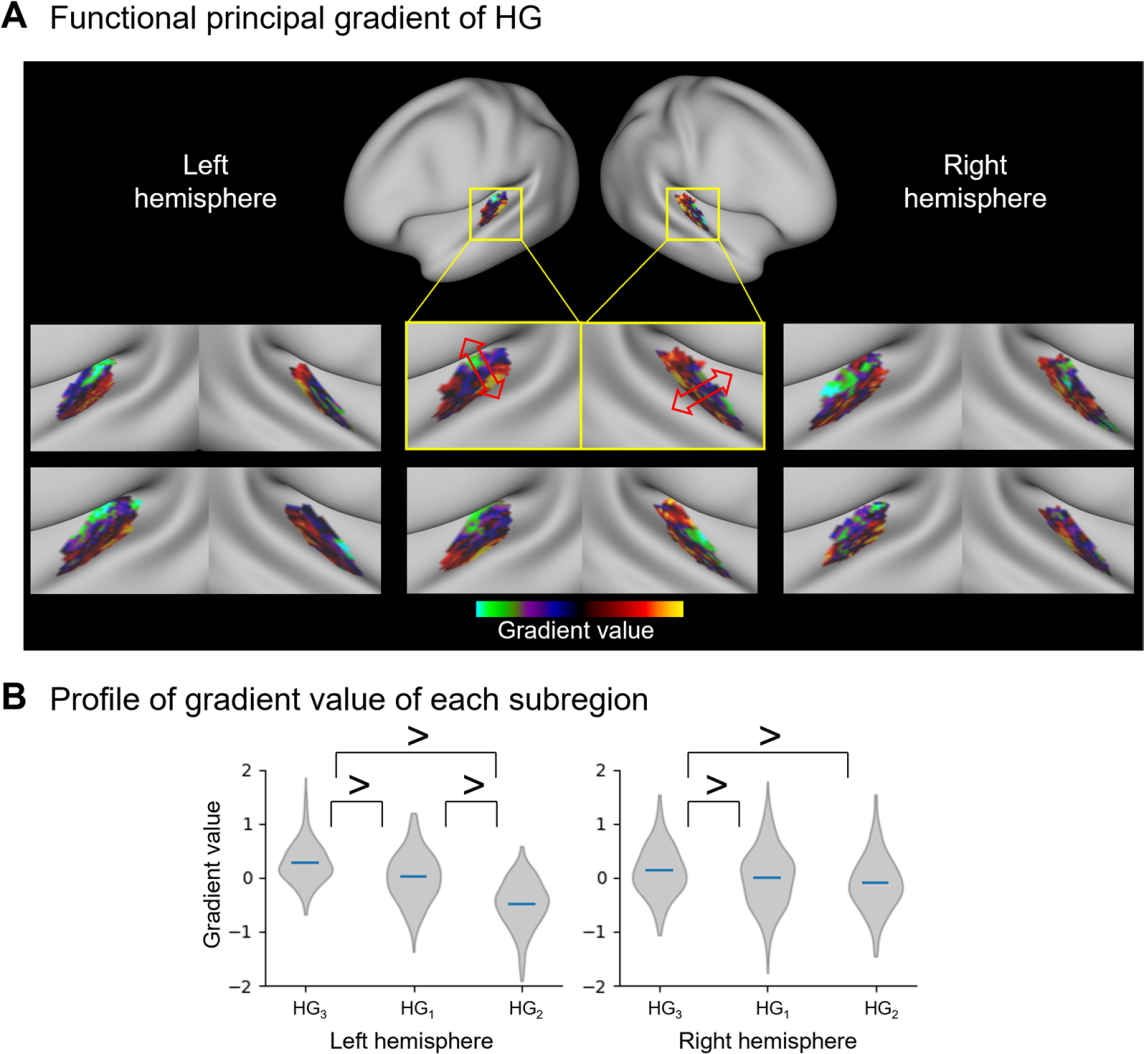
Functional gradient within the HG. (**A**) We assessed the principal gradient within the HG for six randomly selected participants. (**B**) We stratified the gradient value of each subregion, and the significant differences between subregion pairs are indicated by inequality signs.

We used manifold learning to assess whether the HG has a principal axis with gradually changing spatial patterns of functional connectivity. The first functional principal gradient of the HG explained 47.79 $\pm$ 10.41% and 46.14 $\pm$ 10.75% of the variance of the similarity matrix for left and right hemispheres respectively and exhibited a continual axis expanding from the HG_2_ to the HG_3_ (Fig. 4A). This axis is consistent with the relative position of the HG subregions generated using structural connectivity, indicating a correspondence between brain structure and function within the HG. The largest gradient value was observed for the HG_3_, followed by the HG_1_ and then the HG_2_ (p_FWE_ < 0.05), and there was a similar trend between the HG_1_ and HG_2_ in the right hemisphere (p_FWE_ = 0.056; Fig. 4B).

## Discussion

The human auditory cortex is a complex structure consisting of various subregions with distinct functional profiles. By leveraging unsupervised machine learning, we identified three subregions within the HG using diffusion MRI tractography. The subregions exhibited distinct structural and functional connectivity patterns, microstructures, and cortical thickness profiles. Our findings suggest that the human HG can be divided into several subregions, including a highly myelinated central region and less-myelinated lateral and medial areas.

Parcellation is a technique that divides a given brain region into subregions with similar characteristics. Structural connectivity has been used for brain parcellation owing to its robustness and reliability for small structures compared with functional information^12^; thus, we used the structural connectivity derived from diffusion tractography for human auditory cortex parcellation. We found three subregions within the human HG with distinct microstructural and cortical thickness profiles. The central area exhibited a higher degree of myelination and a larger cortical thickness than the other subregions. In previous cytoarchitectural and myeloarchitectural studies, a cortex-wide sensory-fugal gradient was postulatd^45,46^. Additionally, in a recent study on in vivo myelin-sensitive MRI, sensorimotor areas exhibited strong laminar differentiation and high myelination, whereas heteromodal association and paralimbic regions exhibited lower myelin contents and more agranular properties^47^. These findings support our results suggesting that a HG_1_ with a higher myelin content and larger cortical thickness indicates that the central portion of the human auditory cortex is more similar to the brain structures of the primary sensory and motor areas. Thus, the HG_3_ and HG_2_, which exhibited less myelination and a smaller cortical thickness, may be related to higher-order paralimbic regions.

The seed-based functional connectivity analysis complemented our microstructural profile findings, indicating stronger connectivity between the HG_3_ and higher-order cortical areas of the insula, the default mode, and limbic regions than other two subregions. Similarly, the structural connectivity of the HG_3_ with the superior temporal regions was strong. Our findings indicate that the surrounding regions of the HG (not the core area) are involved in the links connected to the heteromodal association and limbic cortices and construct higher-order networks of the brain. In nonhuman primate studies, the belt area situated around the core region exhibited similar properties (less granular structures than the core), and the lateral belt was bordered by the parabelt region, which was located in the STG^3,5^. The core of nonhuman primates partially corresponds to Te1.0 and Te1.1, the lateral belt corresponds to Te1.2 and Te2, and the medial belt corresponds to TI^14,48^; the regions can be distinguished according to the degree of myelination. These studies collectively suggest that subregions of the human HG (i.e., HG_1_, HG_3_, and HG_2_) exhibit structural and functional correspondence to the core, lateral belt, and medial belt regions in nonhuman primates. The subregions of our study were partially consistent with the Te1.0–1.2 in terms of relative positions along the medial-to-lateral direction (Te1.1⟶Te1.0⟶Te1.2 and HG_2_⟶HG_1_⟶HG_3_) and we reported the detailed cluster positions in supplement (Supplementary Fig. 3). In addition, subregions of both hemispheres showed a similar cytoarchitectural pattern including its gradient and lateralization. Cytoarchitectonic studies reported higher cell density in Te1.1 compared to Te1.2 in the left but not in the right hemisphere^14^. Using cortical thickness as a surrogate for cytoarchitectonic feature, we also observed higher cortical thickness of HG_2_ comprared to HG_3_ in the left and vice versa in the right hemisphere. However, we cannot claim correspondence between two sets of subregions because the link between cortical thickness and cytoarchitecture is only demonstrated at the global level but missing at the regional level^49^. Although the superior parietal and orbitofrontal cortices are involved in the default mode and limbic networks, these regions exhibited stronger connections with the HG_1_ and HG_2_ regions than with the HG_3_. This may be because the low temporal signal-to-noise ratios of these regions (Supplementary Fig. 6) led to a low sensitivity and specificity of functional connectivity. To better assess the functional organization and accurately define the boundaries of the subregions, further research using higher-quality 7-T MRI is required.

We used seed-to-whole brain connectivity as the feature to perform clustering for each vertex in the initial ROI. This means we have a 42-element feature that is subject to clustering using the Deskian-Killiany atlas. Using the more modern Destrieux atlas, we need to cluster the 84-element vector because the Destrieux is more fine-grained. The dimension of the feature space is an important parameter in machine learning. With the increased feature dimension (i.e., from the Destrieux atlas), the clustering outcome could become unstable, especially for limited samples (Supplementary Fig. 7). In addition, tractography algorithms could become unstable for small brain regions^50,51^. Due to these two factors, limited feature dimension and instability of tractography, we chose to define the initial ROI based on the relatively coarse-grained Deskian-Killiany atlas.

The most widely used approach for defining the primary auditory cortex in humans is tonotopy, which assesses functional responses to external sounds with different frequencies^52–54^. The group-level tonotopy map revealed that the cell population in the auditory cortex changed according to the frequency range. However, owing to the inter-individual variability of the frequency response, the definitions of the major axes of the human auditory cortex remain under debate (e.g., classical, orthogonal, or with additional regions)^3^. Instead of using tonotopy, we defined a principal axis within the auditory cortex by applying manifold learning techniques to the resting-state functional connectivity, which ran from the HG_1_ to the HG_3_ and HG_2_, consistent with the classical interpretation of tonotopic maps^55,56^. Thus, our gradient approach complements prior works and extends the finding that task-free functional connectivity can be used to identify tonotopy-like spatial patterns within the human HG.

In this study, we divided the human HG into subregions using high-quality structural connectivity information obtained from the HCP database. Unsupervised machine learning revealed structurally and functionally plausible subregions within the HG in humans and their microstructures and tonotopy-like functional gradients provided insights into their underlying topographical properties of the subregions. Further investigations based on high angular resolution diffusion imaging, which allows more accurate modeling of the orientation distribution function in neural fibers than regular diffusion MRI^57^, and multimodal imaging (including tonotopy) may validate our findings.

## Supplementary Information

Supplementary Figures.

## Data Availability

The data used in this study were obtained from the HCP database (https://www.humanconnectome.org/) with approval.

## Code availability

The codes used for the analyses, including the HG parcellation and characterization of structural and functional profiles, are available at https://github.com/hebinalee/human_PAC_parcellation.
