# Supplementary material for: In-vivo data-driven parcellation of Heschl’s gyrus using structural connectivity
Source: Sci Rep. 2022 Jul 4;12:11292. doi: 10.1038/s41598-022-15083-z (PMC9253310; doi:10.1038/s41598-022-15083-z)
Supplement: Supplementary file 1 — Supplementary Figures. [file 41598_2022_15083_MOESM1_ESM.docx]

**Supplementary materials**

**
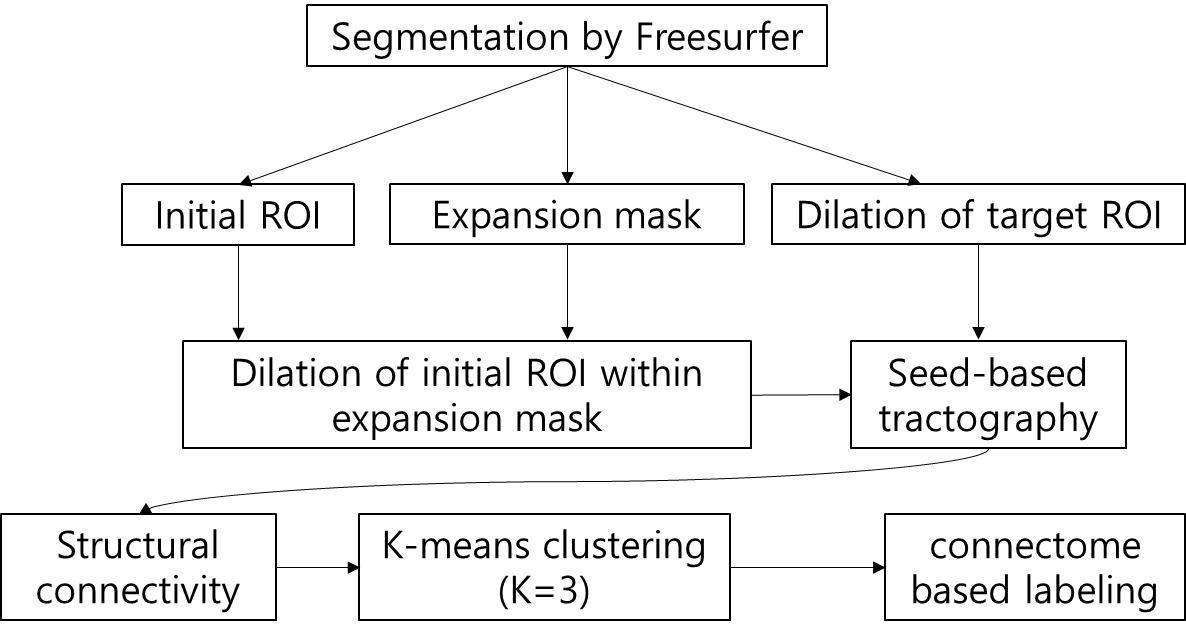
**

**Supplementary Figure 1. A step-by-step protocol to identify subregions.** To perform seed-based tractography, we first specified the source ROI by obtaining the initial ROI (TTG from DK atlas) and then dilated this region by 2.1 mm within the expansion mask. The expansion mask included STG and insula. After seed-based tractography, we calculated structural connectivity between the source ROI and the dilated target ROI. The subregions were obtained using K-means clustering algorithm (with K=3) and were labeled based on structural connectome weights with STG and insula.

*Abbreviations*: DK, Desikan-Killiany atlas; TTG, transverse temporal gyrus; STG, superior temporal gyrus

**
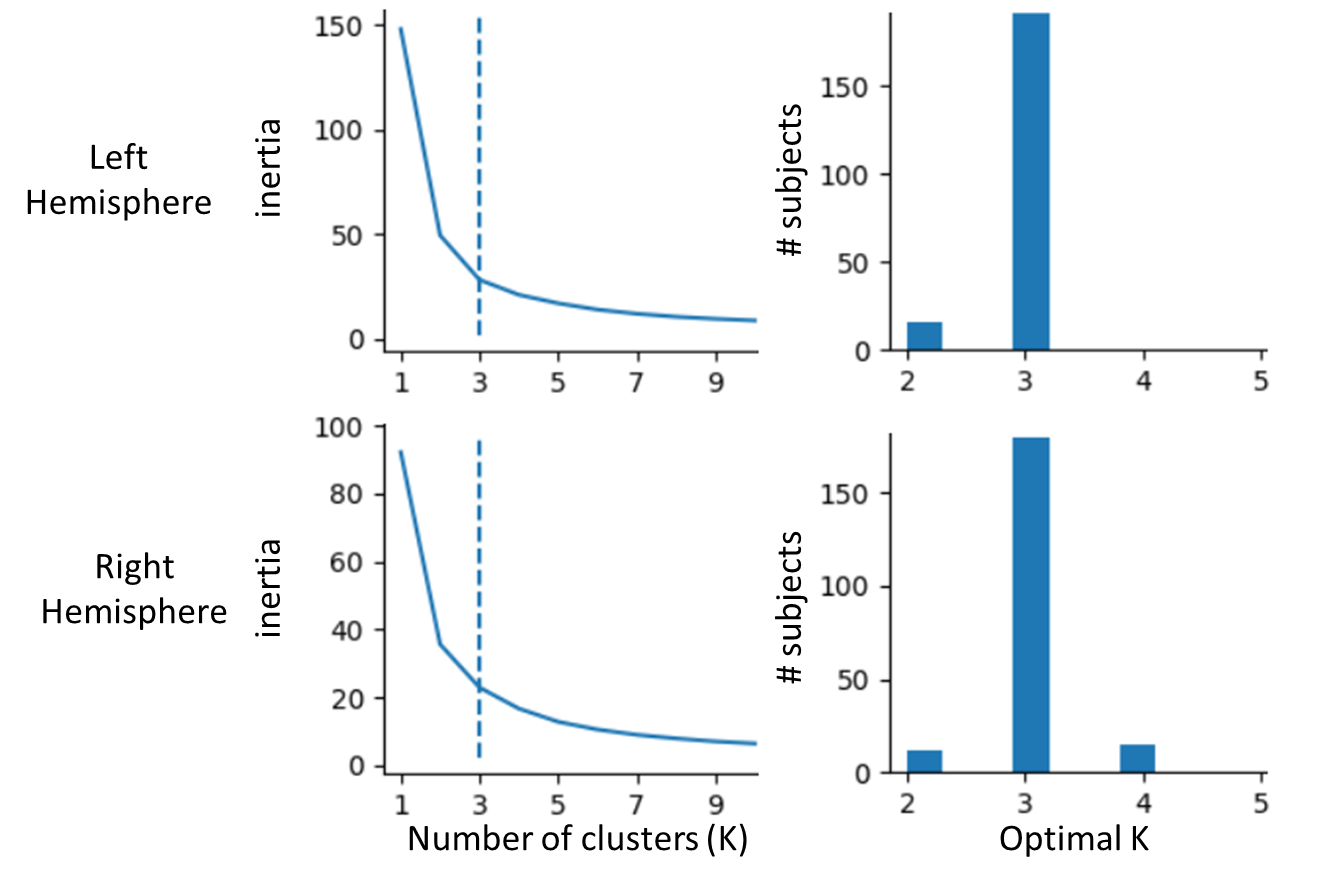
**

**Supplementary Figure 2. The selection of the optimal number of clusters.** We determined the optimal number of clusters by a data-driven approach using a knee point detection algorithm for inertia profile. The left column shows the group averaged inertial profile and its knee point and three was the optimal number detected for each hemisphere. The right column shows the histogram of knee points detected for each subject and the majority was three for both hemispheres.

**
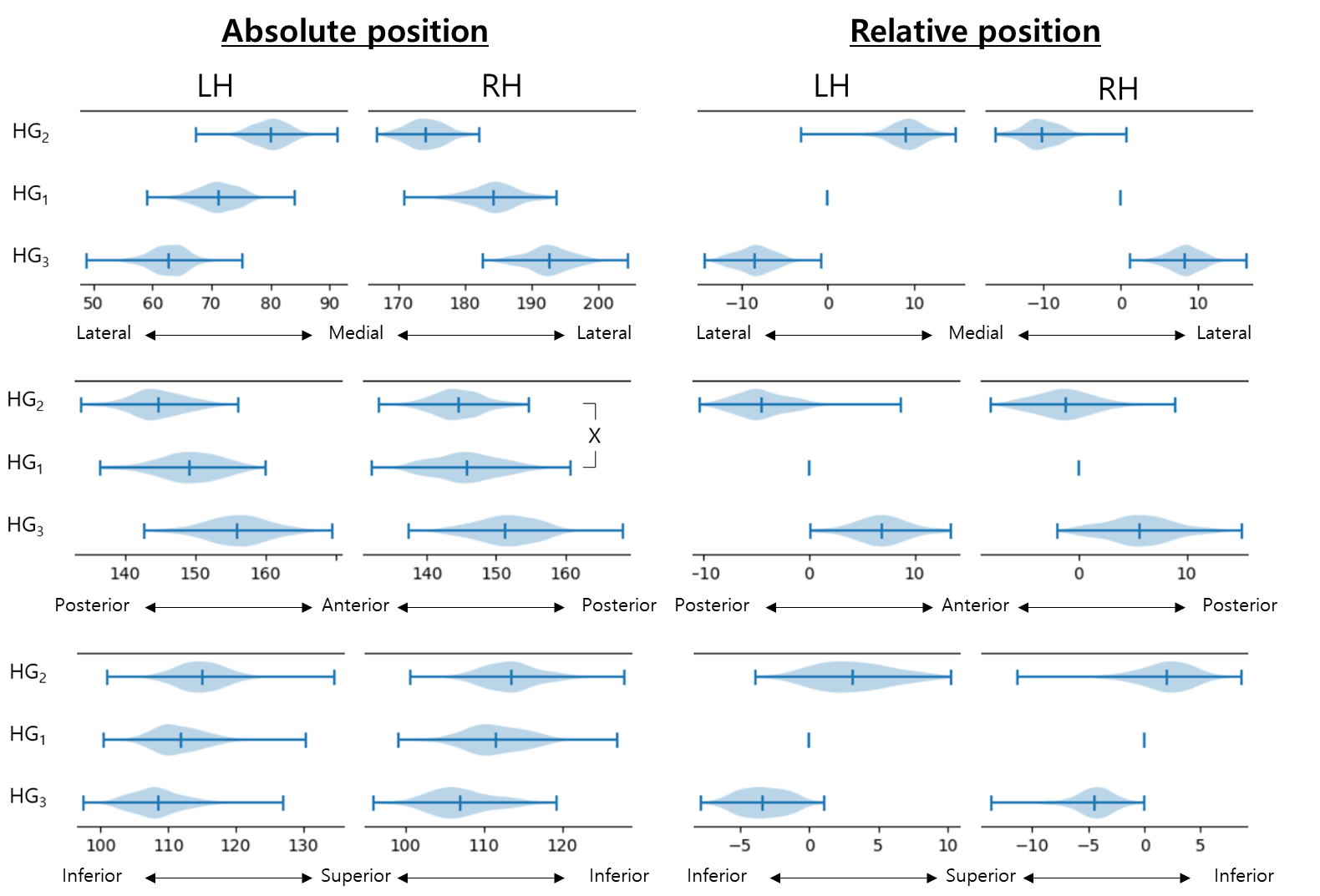
**

**Supplementary Figure 3. Comparison of centroid position for each cluster.** We calculated the centroids of the three clusters and compared them in their absolute and relative positions (distance from the central cluster, HG_1_). Except for comparing HG_1_ and HG_2_ along the anterior-posterior axis (marked as X), all pairs of clusters showed significant position differences (p < 0.05, Bonferroni corrected). HG_1_ was always located between HG_2_ and HG_3_, HG_2_ was the medial-posterior-superior part of HG, and HG_3_ was the lateral-anterior-inferior part of HG for both hemispheres. Especially, three clusters were well separated on the medial-lateral axis.


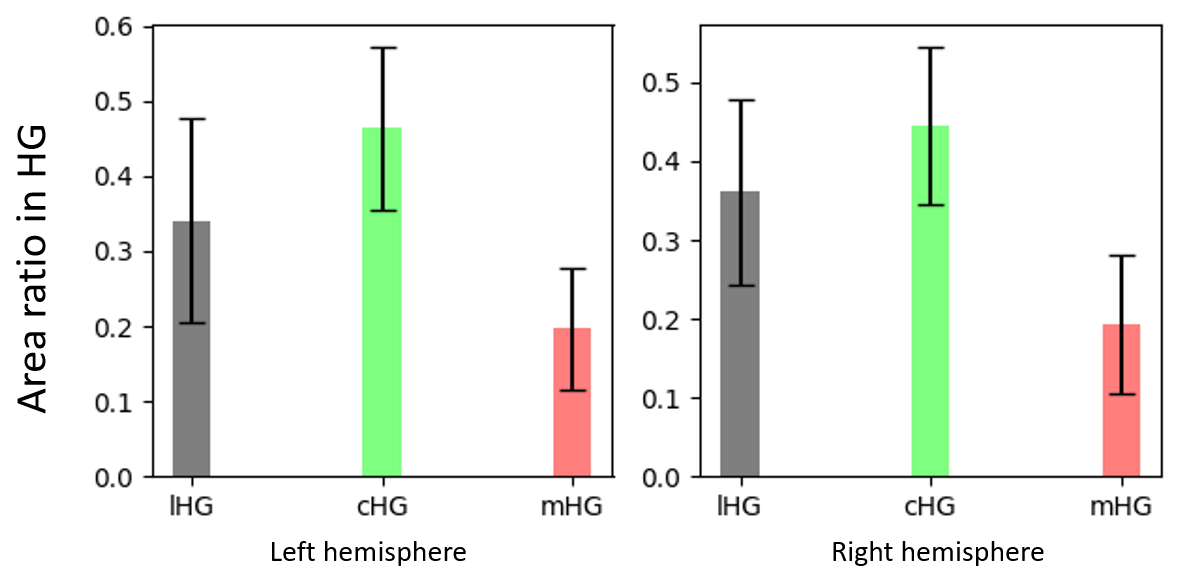


**Supplementary Figure 4. Proportion of each subregion within the HG.** We determined the area ratios by dividing the number of voxels in each subregion by the entire number of voxels in the HG. Error bars indicate the standard deviations across individuals.

**
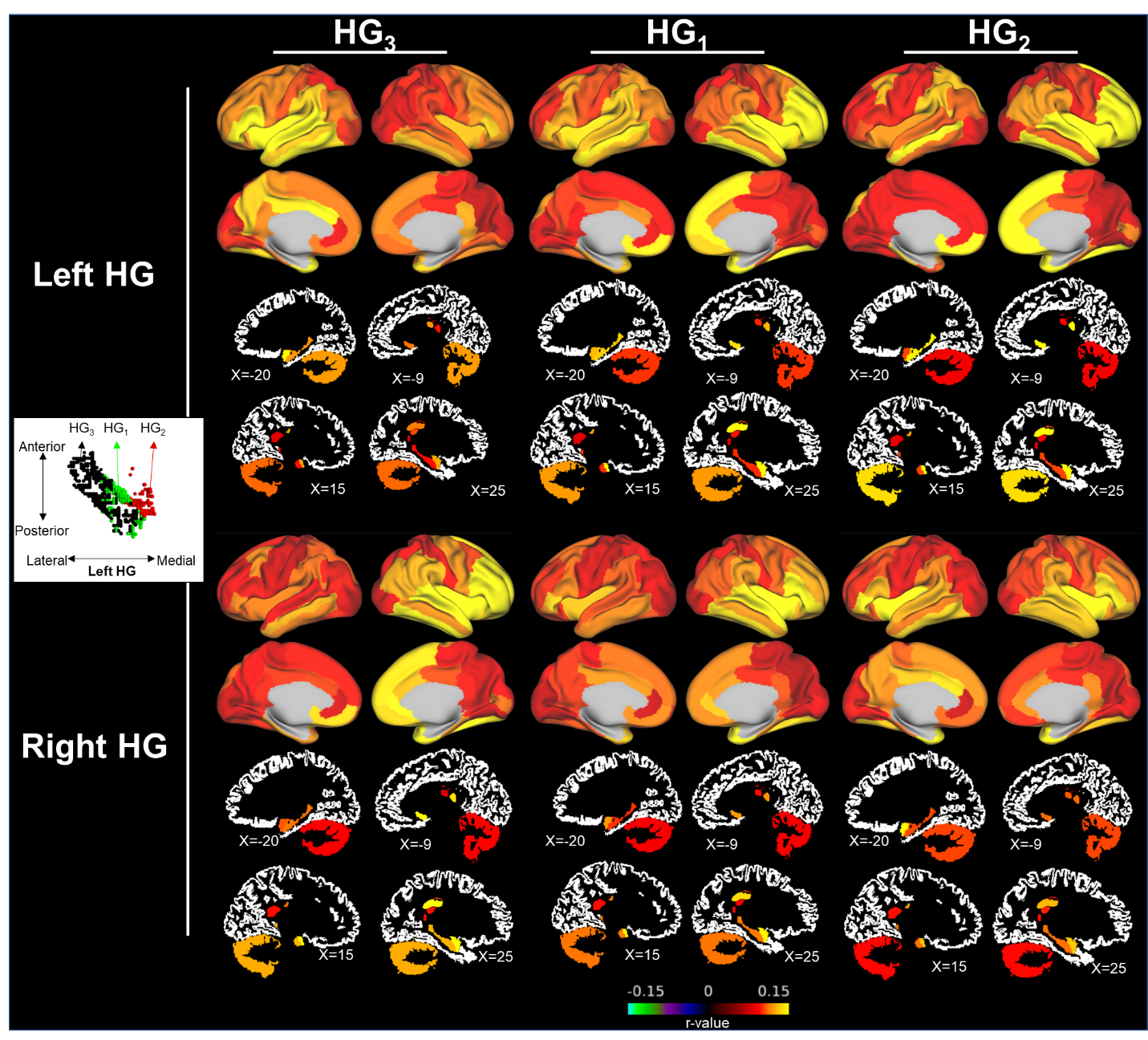
**

**Supplementary Figure 5. The whole-brain seed-based functional connectivity for the three subregions.**


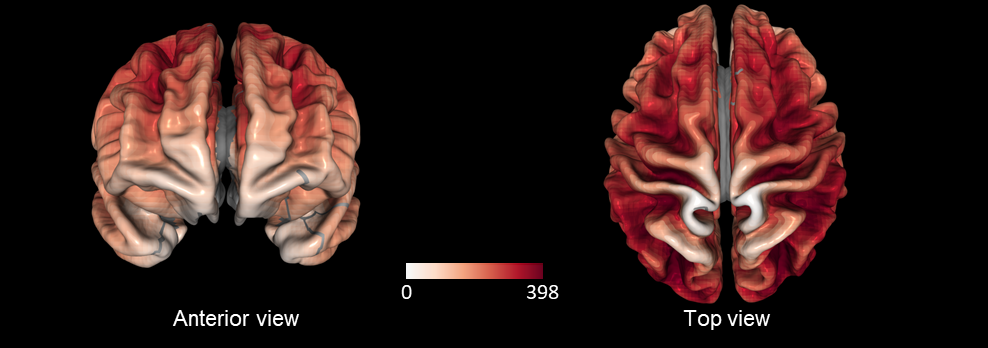


**Supplementary Figure 6. Temporal signal-to-noise ratio (tSNR) map for a semantic task.** The map was provided by NeuroVault (https://identifiers.org/neurovault.collection:8710). The orbitofrontal cortex (anterior view) and superior parietal lobule (top view) exhibited low tSNRs.

**
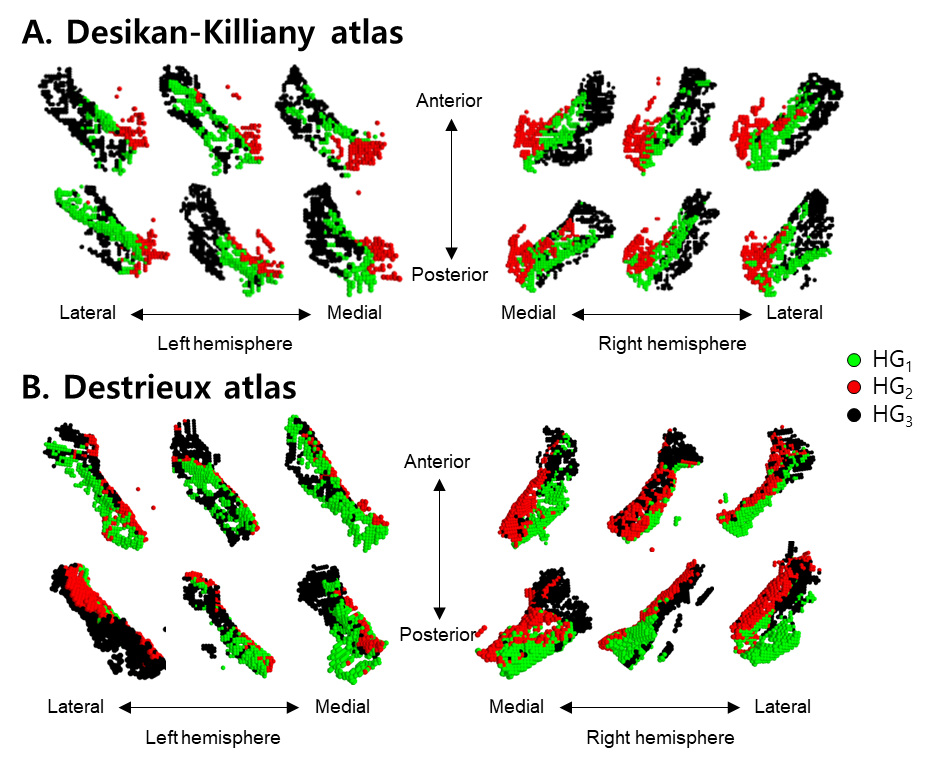
**

**Supplementary Figure 7. Comparison of parcellation results for six participants from Fig. 2.**
